# Evaluation of machine learning algorithms for trabeculectomy outcome prediction in patients with glaucoma

**DOI:** 10.1038/s41598-022-06438-7

**Published:** 2022-02-15

**Authors:** Hasan Ul Banna, Ahmed Zanabli, Brian McMillan, Maria Lehmann, Sumeet Gupta, Michael Gerbo, Joel Palko

**Affiliations:** Department of Ophthalmology and Visual Sciences, West Virginia University School of Medicine, Morgantown, WV 26506 USA

**Keywords:** Surgery, Predictive markers

## Abstract

The purpose of this study was to evaluate the performance of machine learning algorithms to predict trabeculectomy surgical outcomes. Preoperative systemic, demographic and ocular data from consecutive trabeculectomy surgeries from a single academic institution between January 2014 and December 2018 were incorporated into models using random forest, support vector machine, artificial neural networks and multivariable logistic regression. Mean area under the receiver operating characteristic curve (AUC) and accuracy were used to evaluate the discrimination of each model to predict complete success of trabeculectomy surgery at 1 year. The top performing model was optimized using recursive feature selection and hyperparameter tuning. Calibration and net benefit of the final models were assessed. Among the 230 trabeculectomy surgeries performed on 184 patients, 104 (45.2%) were classified as complete success. Random forest was found to be the top performing model with an accuracy of 0.68 and AUC of 0.74 using 5-fold cross-validation to evaluate the final optimized model. These results provide evidence that machine learning models offer value in predicting trabeculectomy outcomes in patients with refractory glaucoma.

## Introduction

Glaucomatous optic neuropathy is the leading cause of irreversible blindness worldwide^1^. The mainstay of glaucoma treatment is lowering intraocular pressure (IOP), which reduces its occurrence and progression^2–4^. Lowering IOP is achieved with ocular topical medications, laser therapy, or incisional surgeries. Incisional surgeries are often necessary for patients with refractory glaucoma who are at high risk for progressive vision loss. Trabeculectomy surgery remains one of the most commonly performed incisional glaucoma surgeries^5^. However, its frequency has declined over the last decade secondary to a growing armamentarium of alternative incisional glaucoma procedures with potentially improved safety profiles^6–9^. The ability to quantify a patient’s risk of failure for a given glaucoma procedure would supplement the shared decision-making process between the patient and physician when determining appropriate treatment plans.

Surgical outcome studies have applied machine learning modeling to predict surgical results for patients undergoing procedures such as corneal refractive surgery, joint replacement and a variety of neurosurgical interventions^10–12^. Yoo et al. found machine learning algorithms statistically superior to classic clinical methods for predicting the complication of corneal ectasia following refractive surgery^10^. Their random forest model had the highest prediction performance of the commonly used machine learning algorithms, with an area under the receiver operating characteristic curve (AUC) of 0.967 on an external validation set. Oermann et al. evaluated several machine learning algorithms to predict morbidity and mortality following stereotactic radiosurgery for cerebral arteriovenous malformation^11^. Their logistic regression model (average AUC 0.71) outperformed existing clinical systems (average AUC 0.63) for predicting poor surgical outcomes at all post-operative time points out to 8 years. Merali et al. applied machine learning to predict quality of life metrics following surgery to treat degenerative cervical myelopathy^12^. Their best performing model utilized a random forest algorithm incorporating neurological exam findings and systemic comorbidities to predict quality of life scores with an AUC of 0.71 at 1 year. These studies highlight the objective of using machine learning to provide outcome predictions at an individual level and advance the field of precision medicine. Like most surgeries, failure of trabeculectomy procedures arises from a complex interaction between many factors. Machine learning may be best suited to model complex non-linear and conditional relationships while generating individual patient-level predictions^13,14^. The objective of this study was to evaluate machine learning models in their ability to predict real world trabeculectomy outcomes using readily available preoperative patient demographic, ocular and systemic health data.

## Results

**Figure 1:**
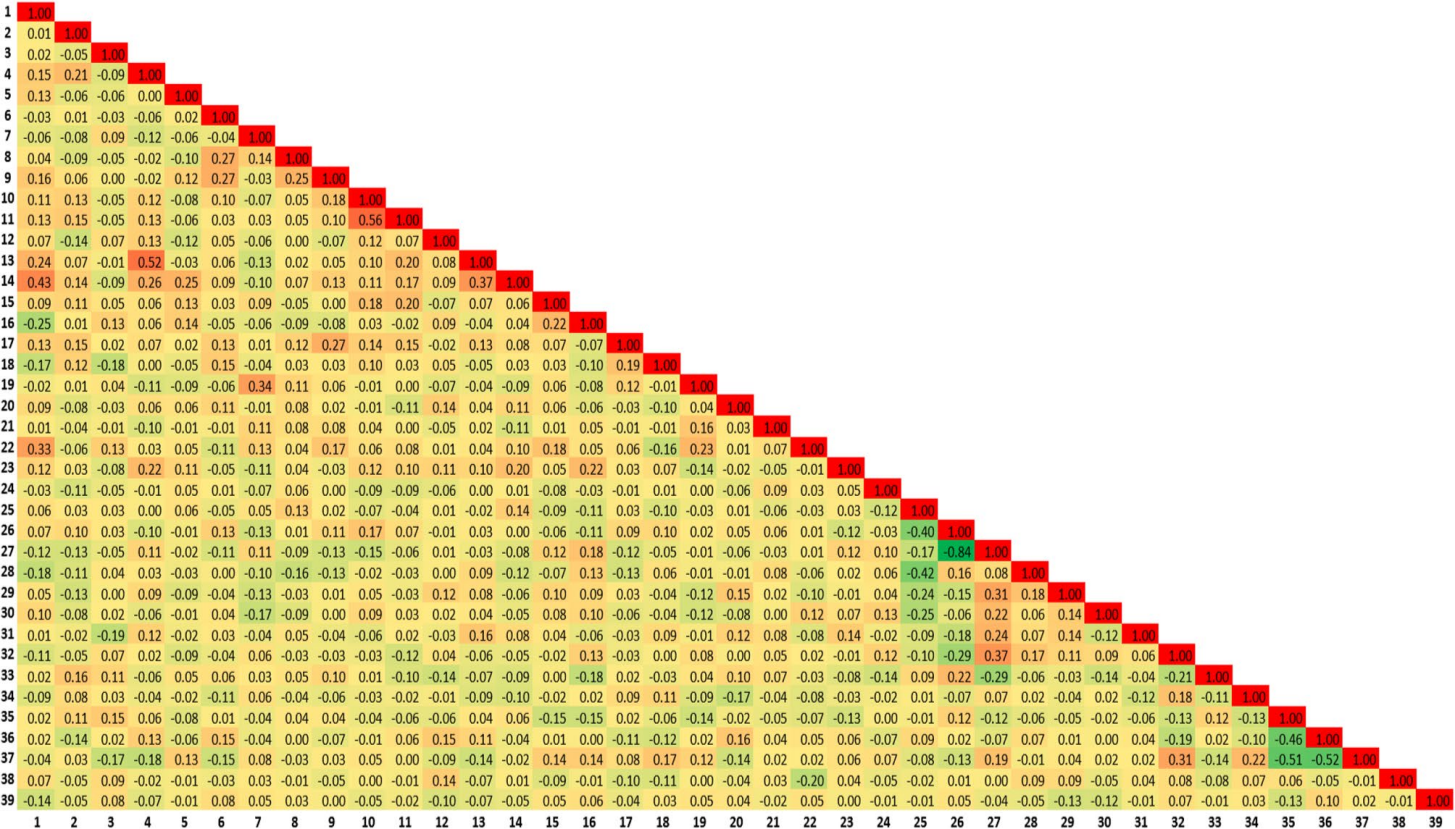
Pairwise pearson correlation map of features. The feature number corresponds to its number as presented in Tables 1, 2 and 3.

Of the 296 consecutive trabeculectomy procedures performed, 230 were performed on 184 patients and included in our analysis based on our exclusion criteria. At 1 year, a total of 104 (45.2%) eyes were classified as complete successes and 126 (54.78%) as surgical failures. A total of 35 preoperative parameters were collected for model input consisting of 3 demographic parameters, 15 parameters from systemic health data and 17 ocular parameters. Six dummy variables were used to transform preoperative IOP magnitude and number of topical glaucoma medications into grouped categorical features. Continuous features included age, body mass index (BMI), preoperative visual acuity (VA) and central corneal thickness (CCT). No feature was found to have a significant positive correlation with any other as shown in Fig. 1. The systemic, demographic and ocular (SDO) dataset consisted of 39 features and the demographic and ocular (DO) dataset 24 features. Tables 1, 2 and 3 show baseline characteristics of both surgical success and failure groups. On univariate analysis, no statistically significant differences were observed in demographic and ocular features between the success and failure groups. A history of myocardial infarction (MI) was the only systemic health feature with a statistically significant difference between groups on univariate analysis (*P* = 0.045).

**Table 1:** Univariate analysis of demographic features recorded in the electronic health record system.

Features	Surgical failure (n = 126)	Surgical success (n = 104)	*P* value
(1) Age (Mean, SD)	69.24 (12.19)	68.08 (12.55)	0.479
(2) Gender			0.678
Male (n, %)	76 (60.3)	59 (56.7)	
Female (n, %)	50 (39.7)	45 (43.3)	
(3) Race			0.066
White (n, %)	110 (87.3)	99 (95.2)	
Black (n, %)	16 (12.7)	5 (4.8)	

n = number; SD = standard deviation.

The threshold for statistical significance was *P* $< .05$.

**Table 2:** Univariate analysis of systemic features recorded in the electronic health record system.

Features	Surgical failure ($\hbox {n}=126$)	Surgical success ($\hbox {n}=104$)	*P* value
**Systemic Meds:**			
(4) Statins (n, %)	67 (53.2)	54 (51.9)	0.955
(5) Angiotensin II receptor blockers (ARBs) (n, %)	22 (17.5)	27 (26.0)	0.160
(6) Inhaled corticosteriods (n, %)	10 (7.9)	15 (14.4)	0.174
(7) Nonsteroidal immunosuppressants (n, %)	10 (7.9)	6 (5.8)	0.702
**Comorbid Conditions:**			
(8) Asthma (n, %)	13 (10.3)	9 (8.7)	0.840
(9) Chronic obstructive pulmonary disease (COPD) (n, %)	8 (6.3)	14 (13.5)	0.110
(10) Congestive heart failure (CHF) (n, %)	9 (7.1)	4 (3.8)	0.429
(11) Myocardial infarction (MI) (n, %)	17 (13.5)	5 (4.8)	0.045
(12) Cerebrovascular accident (CVA) (n, %)	4 (3.2)	7 (6.7)	0.343
(13) Hyperlipidemia (HLD) (n, %)	66 (52.4)	62 (59.6)	0.334
(14) Hypertension (HTN) (n, %)	91 (72.2)	76 (73.1)	1.000
(15) Obstructive sleep apnea (OSA) (n, %)	13 (10.3)	8 (7.7)	0.647
(16) Body Mass Index (BMI) (Mean, SD)	29.1 (6.8)	28.9 (6.8)	0.858
(17) Smoking history (n, %)	52 (41.3)	43 (41.3)	1.000
(18) Active smoker (n, %)	13 (10.3)	12 (11.5)	0.934

n = number; SD = standard deviation.

The threshold for statistical significance was *P* $< .05$.

**Table 3:** Univariate analysis of ocular features recorded in the electronic health record system.

Features	Surgical failure ($\hbox {n}=126$)	Surgical success ($\hbox {n}=104$)	*P* value
(19) History of angle surgery (n, %)	11 (8.7)	9 (8.7)	1.000
(20) History of selective laser trabeculoplasty (SLT) (n, %)	44 (34.9)	33 (31.7)	0.712
(21) History of previous trabeculectomy (n, %)	5 (4.0)	5 (4.8)	1.000
(22) Pseudophakia (n, %)	43 (34.1)	36 (34.6)	1.000
(23) Diabetes mellitus (DM) (n, %)	36 (28.6)	35 (33.7)	0.492
(24) Study eye			1.000
Left (n, %)	63 (50.0)	52 (50.0)	
Right (n, %)	63 (50.0)	52 (50.0)	
**Number of pre-operative topical glaucoma medications**			
(25) (0, 1) (n, %)	7 (5.6)	10 (9.6)	0.359
(26) (2, 3) (n, %)	82 (65.1)	71 (68.3)	0.712
(27) (4, 5) (n, %)	37 (29.4)	23 (22.1)	0.273
(28) Topical beta blocker (n, %)	92 (73.0)	74 (71.2)	0.868
(29) Topical prostaglandin analogue (PGA) (n, %)	103 (81.7)	73 (70.2)	0.057
(30) Topical alpha-agonist (n, %)	62 (49.2)	53 (51.0)	0.895
(31) Topical carbonic anhydrase inhibitor (CAI) (n, %)	67 (53.2)	51 (49.0)	0.623
(32) Oral CAI (n, %)	17 (13.5)	10 (9.6)	0.482
(33) Angle anatomy			0.647
Open angle glaucoma (OAG) (n, %)	113 (89.7)	96 (92.3)	
Angle closure glaucoma (ACG) (n, %)	13 (10.3)	8 (7.7)	
(34) Pre-operative visual acuity (VA) (Mean, SD)	0.49 (0.6)	0.46 (0.7)	0.720
**Pre-operative IOP**			
(35) < 18 mmHg (n, %)	42 (33.3)	30 (28.8)	0.557
(36) $>=$ 18 and $<= 25$ (n,%)	40 (31.7)	34 (32.7)	0.991
(37) $> 25$ (n,%)	44 (34.9)	40 (38.5)	0.676
(38) Surgery-trabeculectomy with cataract extraction (n, %)	21 (16.7)	13 (12.5)	0.484
(39) Central corneal thickness (CCT) (Mean, SD)	554.9 (48.3)	548.8 (45.2)	0.326

$$\hbox {n}= \hbox {number}; \hbox {SD}= \hbox {standard deviation}$$.

The threshold for statistical significance was *P* $< .05$.

**Figure 2:**
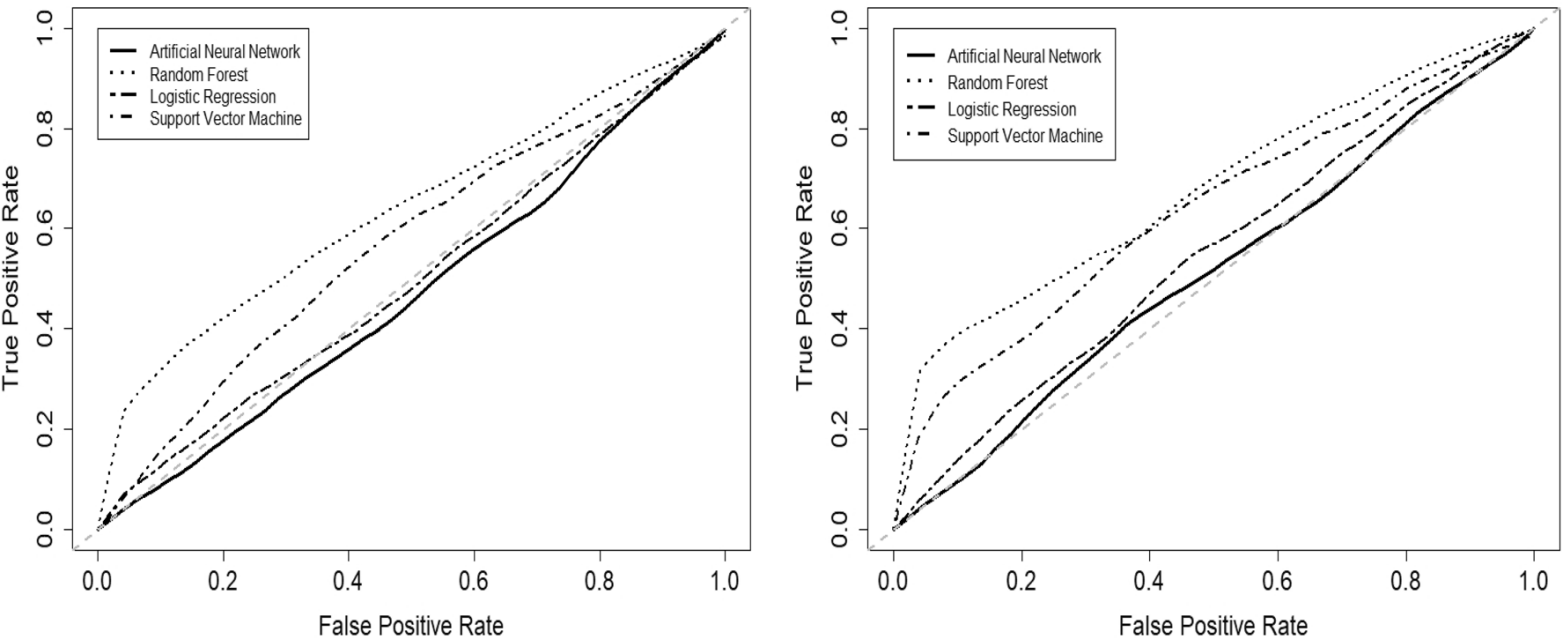
Average receiver operating characteristic curves for the 4 predictive models trained on (left) demographic and ocular (DO) dataset and (right) systemic, demographic and ocular (SDO) dataset.

The performance of the four predictive models evaluated with 5-fold cross validation are shown in Table 4 for the DO dataset and Table 5 for the SDO dataset. Random forest (RF) provided the highest accuracy, with values of 0.64 and 0.65 for the DO and SDO datasets, respectively. The average receiver operating characteristic curves for each model are shown in Figure 2 for both DO and SDO datasets. Random forest also showed the highest mean area under receiver operating characteristic curve (AUC), with values of 0.64 and 0.68 for the DO and SDO datasets, respectively. The RF model had the lowest sensitivity and highest specificity compared to support vector machine (SVM), logistic regression (LR) and the artificial neural network (ANN).

Table 6 lists the relative contribution of various predictor features from the SDO dataset for the LR model. Features associated with significantly increased risk of trabeculectomy failure in the LR model were use of preoperative statin therapy (OR = 0.74, *P* = 0.045), preoperative topical prostaglandin analogue (PGA) therapy (OR = 0.52, *P* = 0.041), a history of MI (OR = 0.32, *P* = 0.032) and male gender (OR = 0.31, *P* = 0.023). White race (OR = 2.88, *P* = 0.046) was significantly associated with trabeculectomy success in the LR model.

**Figure 3:**
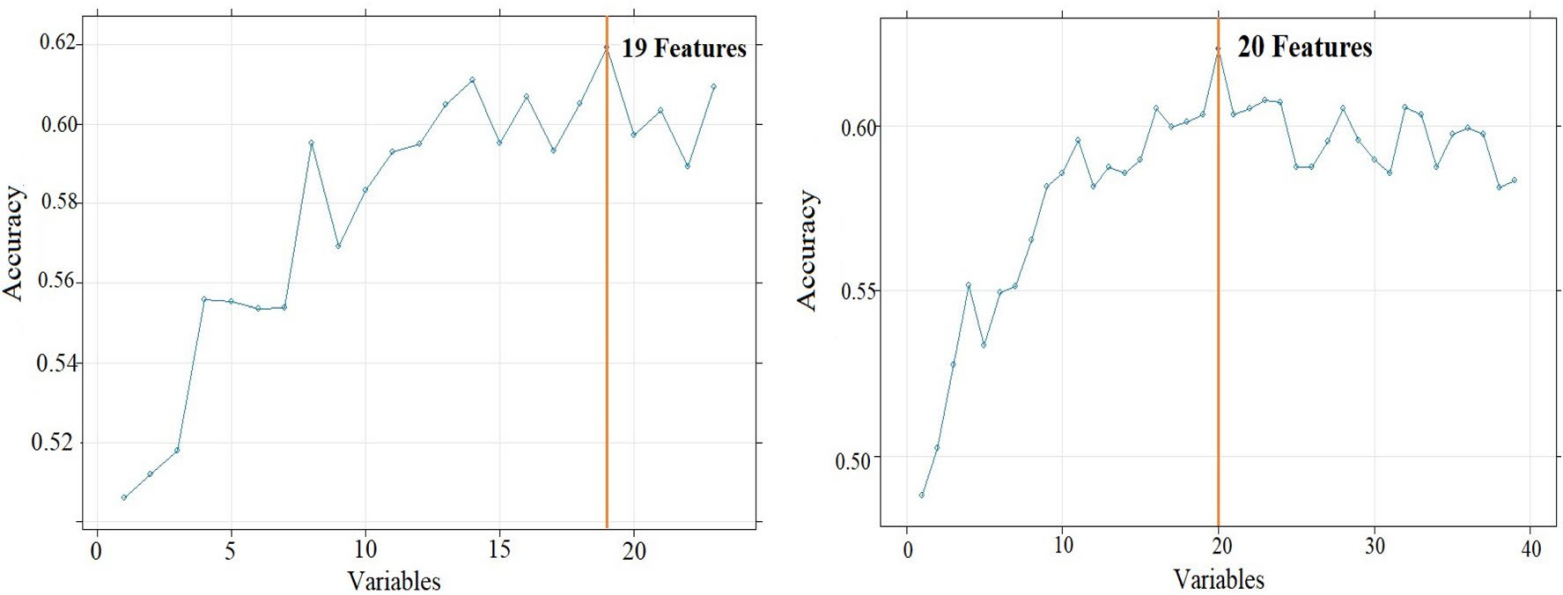
Results of recursive feature elimination algorithm applied to the random forest model using the (left) demographic and ocular (DO) dataset and (right) systemic, demographic and ocular (SDO) dataset.

The random forest model was chosen for further optimization secondary to its greater accuracy and AUC on initial evaluation of the models. Feature selection using recursive feature elimination was applied to all 39 features from the SDO dataset and all 24 features from the DO dataset. This process resulted in 19 features for the DO dataset and 20 features for SDO dataset as shown in Fig. 3. After feature selection, hyperparameter tuning was performed on the RF model using a grid search scheme varying “mtry” and “number of trees”. The final optimized random forest model had 500 trees with a “mtry” of 2.

**Table 4:** Comparison of predictive models trained using the the demographic and ocular (DO) dataset.

Predictive model	Accuracy	Sensitivity	Specificity	AUC
Random forest	0.64	0.52	0.77	0.64
Logistic regression	0.54	0.55	0.53	0.50
Artificial neural network	0.56	0.60	0.51	0.47
Support vector machine	0.59	0.57	0.62	0.57

AUC = area under the receiver operating characteristic curve.

**Table 5:** Comparison of predictive models trained using the systemic, demographic and ocular (SDO) dataset.

Predictive model	Accuracy	Sensitivity	Specificity	AUC
Random forest	0.65	0.44	0.86	0.68
Logistic regression	0.59	0.58	0.57	0.55
Artificial neural network	0.53	0.49	0.59	0.51
Support vector machine	0.62	0.48	0.76	0.64

AUC = area under the receiver operating characteristic curve.

**Table 6:** Relative contribution of various features in the multivariate logistic regression model predicting outcomes of trabeculectomy surgical intervention.

Features	Odds ratio (95% confidence interval)	*P* value
Male gender	0.31 (0.10, 0.79)	0.023
Topical prostaglandin analogue (PGA)	0.52 (0.28, 0.97)	0.041
Myocardial infarction (MI)	0.32 (0.10, 0.85)	0.032
Systemic Meds: Statins	0.74 (0.54, 0.99)	0.045
White race	2.88 (1.08, 9.06)	0.046

The threshold for statistical significance was *P* $< .05$.

**Figure 4:**
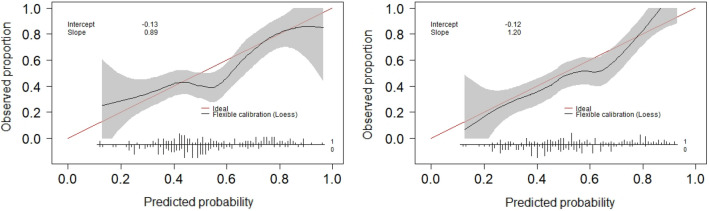
Calibration curves for the optimized random forest models using the (left) demographic and ocular (DO) and (right) systemic, demographic and ocular (SDO) datasets. The flexible curve with pointwise confidence intervals (gray area) was based on local regression (loess). The bottom of the graph shows histograms of the predicted risks for eyes with complete success (1) and failure (0) at 1 year.

**Figure 5:**
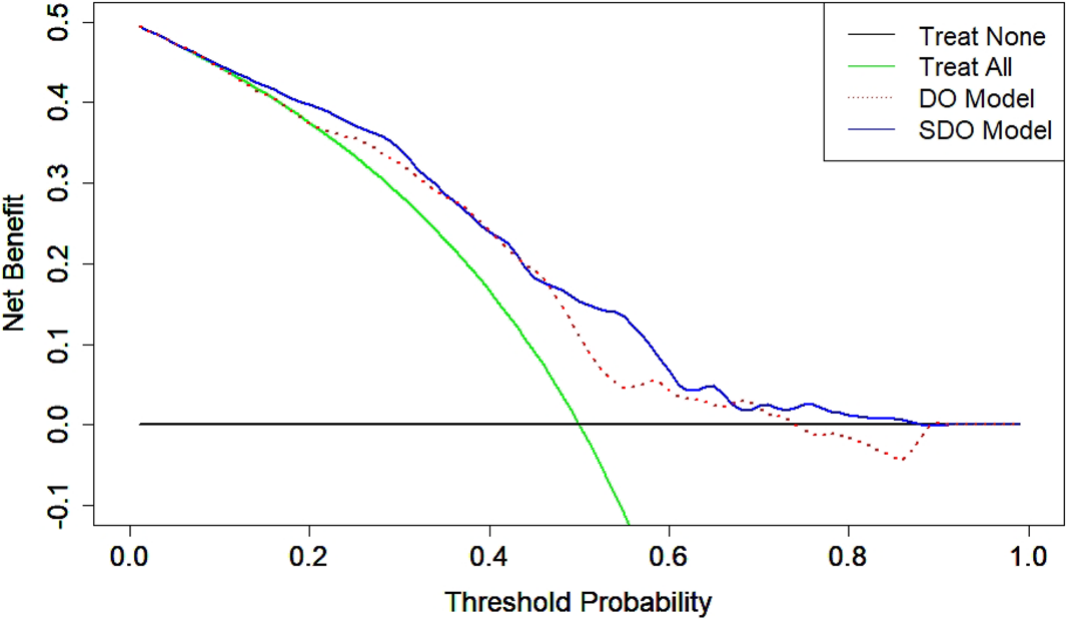
Decision curve analysis (DCA) for the optimized random forest models. The model trained on the SDO dataset trended towards greater net benefit compared to the DO trained model. Both models provided a greater net benefit compared to the decision of performing surgery on all or no patients across a wide range of threshold probabilities.

**Figure 6:**
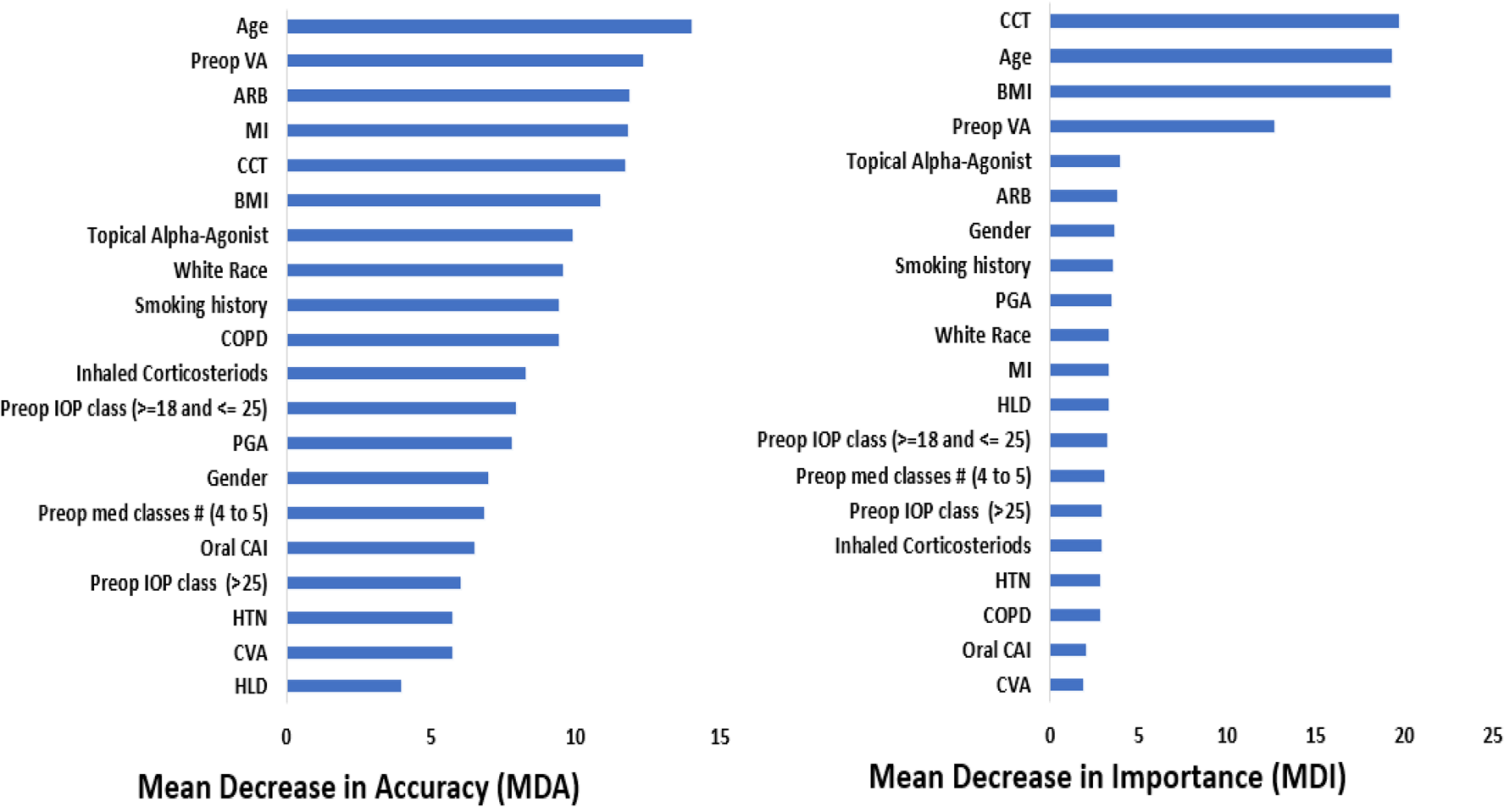
Importance of top features from the systemic, demographic and ocular (SDO) dataset based on (left) MDA (right) MDI.

The performance of the optimized RF model was evaluated using 5-fold cross validation on the DO and SDO datasets. The model predicted trabeculectomy surgical outcomes with an accuracy of 0.67 and 0.68 and with a mean AUC of 0.68 and 0.74 for the DO and SDO datasets, respectively. Additional discrimination metrics such as sensitivity, specificity, positive predictive value (PPV) and negative predictive value (NPV) for the two models are listed in Table 7. The calibration curves for the DO and SDO random forest models are shown in Fig. 4 with corresponding slopes and intercepts. The decision curve analysis (DCA) plots for the final DO and SDO random forest models are shown in Fig. 5. Figure 6 shows the most important predictive features of the optimized RF model from the SDO dataset.

**Table 7:** Comparison of the optimized random forest models trained on the demographic and ocular (DO) features and systemic, demographic and ocular (SDO) features.

Case study	Accuracy	Sensitivity	Specificity	AUC	PPV	NPV
DO features	0.67	0.49	0.86	0.68	0.71	0.64
SDO features	0.68	0.60	0.76	0.74	0.69	0.65

AUC = area under the receiver operating characteristic curve; PPV = positive predictive value; NPV = negative predictive value.

## Discussion

To our knowledge, this is the first study to leverage the use of machine learning algorithms to predict trabeculectomy outcomes. In this retrospective study, we developed and compared different machine learning models utilizing preoperative patient data available in electronic health records (EHR) to predict 1 year complete success of consecutive trabeculectomy surgeries. The datasets included features from traditional ocular and demographic variables in addition to readily accessible systemic health data. The RF model marginally outperformed LR, ANN and SVM models using AUC and accuracy as evaluation metrics. Models trained with the full SDO dataset moderately outperformed models trained with the DO dataset in discrimination and net benefit. The performance of our models supports the hypothesis that machine learning models using preoperative features, including systemic health data, has the potential to aid physicians and patients in surgical decision making.

Several studies have investigated the influence of patient and ocular features on trabeculectomy surgical outcomes^15–21^. These studies have found younger age, black race, previous ophthalmic procedures, worse preoperative VA, preoperative IOP magnitude, greater number of preoperative IOP lowering topical medications and a history of diabetes mellitus as risk factors for trabeculectomy failure. In our logistic regression model, male gender, the use of preoperative PGA drops, history of MI and use of statin therapy were associated with a significantly increased risk of trabeculectomy failure while white race was associated with trabeculectomy success at 1 year. Although more difficult to interpret compared to LR models, our RF model showed age, preoperative VA, use of angiotensin II receptor blockers (ARBs), history of MI, CCT and BMI as the most important features in predicting trabeculectomy surgical outcomes using MDA. Using MDI, CCT, age, BMI, and preoperative VA were the most important features. The RF model provides new insights into potential factors that may influence trabeculectomy outcomes.

Our optimized RF model had an accuracy of 0.68 and AUC of 0.74 using the SDO dataset. We considered this a reasonable outcome given the complexity of the physiology and smaller sample size. Using machine learning algorithms to predict complications in deep brain stimulation surgery, a gradient boosting algorithm predicted surgical complications with an accuracy of 0.66 and AUC of 0.58 when the model was applied to their original dataset^22^. Lei et al applied machine learning to predict acute kidney injury after aortic arch surgery with an AUC of 0.71 using a RF model^23^. Rahman et al predicted recurrence after esophageal cancer with an AUC of 0.805 using a RF model^24^. Our classes were relatively balanced (complete success class was 45.2% of total sample) compared to other surgical outcome studies using machine learning to predict more rare surgical outcome events^22,24–26^. It is important to note that we chose a clinically stringent definition of surgical failure to maintain more balanced classes. Our qualified success rate, which includes complete successes and eyes which met the definition of complete success but were also on supplemental medical therapy to lower IOP, was 71.3%. Another strength of the current study was the complete feature data for each patient, obviating the common practice of generating synthetic values for missing data^27,28^.

The RF model moderately outperformed SVM, LR and ANNs models. Other studies have shown similar performance results for classification tasks using machine learning models on healthcare datasets^12,29^. This is likely due to the intrinsic nature of these models to learn non-linear complex relationships that may be missed by LR models. The poorer performance of ANNs is suspected to be related to the small sample size available to train the ANN, since ANNs usually require larger training datasets than RF or SVM. The improved accuracy and AUC of RF compared to SVM is likely due to the ability of RF models to avoid over-fitting on datasets with low sample to feature ratios^12^. Our dataset has approximately 5:1 sample to feature ratio, which can limit the convergence of SVM to a local minimum. A low sample to feature ratio is a common barrier of machine learning in the medical field and thus RF can be a good choice for classification tasks such as the one considered in this paper.

We evaluated the ’weak calibration’ of the final DO and SDO random forest models^30^. The slopes of the calibration curves were 1.20 and 0.89 for the SDO and DO random forest models, respectively. The calibration slope evaluates the spread of the estimated risks, indicating that the DO model risk estimates are slightly extreme (i.e., too high for eyes that are high risk and too low for eyes at low risk) and SDO estimates more moderate. The intercepts of the calibration curves were − 0.12 and − 0.13 for the SDO and DO models, respectively. The calibration intercepts of the models, which assesses calibration-in-the-large, suggest that the both the SDO and DO models overestimate risk somewhat. The DCA provides complimentary model information which may help in the decision making process of whether to proceed with trabeculectomy surgery or try alternative treatment strategies. The DCA of the SDO trained model showed a positive net benefit compared to treat-all or treat-no eyes schemes across a threshold probability range from 0.06 to 0.89.

There were several limitations to the current study. First, the small sample size with a white to black ratio of approximately 10 to 1 collected from a single tertiary teaching hospital in a retrospective manner limits the generalization of the model to other populations. Prospective evaluation of the model on other datasets are necessary to determine if the model translates into benefits for future patients. Second, 21.9% of patients were lost to follow up at 1 year which likely introduces bias into this retrospective cohort. As many of these patients were referred back to their local eye care provider, it is quite possible that the drop out population had a higher complete success rate if not referred back to our institution for further management. This may partially explain the relatively lower percentage of complete success (45.21%) in our cohort compared to previous trabeculectomy outcome studies, in addition to our liberal inclusion of eyes with all glaucoma subtypes, previous ocular surgery, and the high proportion with a preoperative IOP less than 18 mmHg (31.3%)^6^. Third, our model was designed for single layer prediction of success or failure with no ability to predict the cause of failure (i.e., hypotony vs lack of sufficient IOP lowering). Future work will evaluate the ability of stacking machine learning approaches to further predict the cause of trabeculectomy failure^31^. Finally, our study included 230 trabeculectomy surgeries performed on 184 patients, with 46 patients receiving bilateral trabeculectomies, which may have lead to some data leakage. We also analyzed the performance of the optimized RF model by considering only one trabeculectomy surgery from each patient. The accuracy and AUC for the SDO features in this subset was 0.66 and 0.70, respectively, with a small reduction in accuracy and AUC as compared to the full dataset.

Despite these limitations, we believe this study is an important initial step in evaluating machine learning models to predict glaucoma surgical outcomes. We have shown that machine learning models offer value in predicting trabeculectomy success and the integration of systemic health data in additional to standard ophthalmic and demographic data can improve model performance. As surgical options in glaucoma expand, predictive models have the potential to improve patient care and aid in the surgical decision making process. Future work will focus on utilizing these algorithms with a larger dataset, such as those that can be provided by the Sight Outcomes Research Collaborative (SOURCE) Ophthalmology Data Repository^32^.

## Methods

### Patient population

Patient data was obtained retrospectively from consecutive adult patients undergoing trabeculectomy or trabeculectomy and cataract extraction with intraocular lens implantation from January 2014 to January 2018 at the West Virginia University (WVU) Eye Institute. Approval from the WVU Institutional Review Board through the office of human research protections to collect patient data with a waiver of informed consent was obtained prior to data collection. All research adhered to the tenets of the Declaration of Helsinki and was compliant with the Health Insurance Portability and Accountability Act. Data was collected for each patient via chart review from the hospital electronic health record EPIC (Epic Systems, Verona, WI) by fellowship trained glaucoma surgeons (JP, SG, and BM). The features and outcomes data were then reviewed for completeness and accuracy (JP), and organized within a secure tabular data sheet. No outliers were removed from the dataset and no missing-value management was required. Data collected included preoperative systemic health data, preoperative and postoperative ocular data and demographic data. All patients were 18 years or older with primary open-angle, pseudoexfoliation, pigment dispersion, juvenile and chronic primary angle closure glaucoma. Exclusion criteria included patients under 18 years of age, those that received an Express shunt (Alcon, Forth Worth, Texas, USA) and patient with less than 1 year of follow up at our institution.

### Trabeculectomy surgical technique

All patient surgeries were completed by or with the guidance of a fellowship trained glaucoma surgeon at our institution’s outpatient surgical center. All trabeculectomies were performed in a similar manner. A subconjunctival injection of 1% lidocaine mixed with 40 mcg of mitomycin C (0.2 mg/mL) was injected superior prior to conjunctival incision. A fornix-based conjunctival flap, partial thickness rectangular scleral flap and sclerostomy were created. A peripheral iridectomy was performed in all phakic eyes and in pseudophakic eyes with any iris prolapse. The scleral flap was closed tight with interrupted 10-0 nylon sutures to allow flow only with supraphysiologic IOP. The conjunctiva was reapposed with 8-0 polyglactin interrupted wing sutures. All patients received antibiotic drop prophylaxis for 1 week and steroid drops for a minimum of 4 weeks postoperatively. Selective laser suture lysis was performed in the postoperative period for IOP titration.

### Baseline features, outcome classification and class balancing

Criteria for selecting input features for the models included existing evidence in the literature suggesting a relationship between the feature and the surgical outcome, clinical domain knowledge and availability of the feature in the dataset. Baseline features included patient demographic and systemic health data (e.g., co-morbidities, chronic medications, smoking status, etc.). Preoperative ocular data included prior ocular history, current glaucoma drops and relevant exam data at the appointment in which the decision was made to proceed with trabeculectomy surgery. A full list of preoperative features are shown in Tables 1, 2 and 3. The primary outcome classification was surgical failure of trabeculectomy at the 1 year postoperative visit. Surgical failure was defined as IOP $> 21$ mmHg or $< 5$ mmHg at two consecutive visits after 3 months, less than a 20% IOP reduction at two consecutive visits after 3 months, a need for reoperation for glaucoma or loss of light perception vision. Eyes which had not failed by the above criteria and were not receiving supplemental medical therapy to lower IOP were considered complete success and the remaining eyes considered failures. Postoperative manipulations of the trabeculectomy site (e.g., needling revisions) were allowed in the success class if the patient did not require a return to the operating room. Postoperative manipulations were not included as features given the goal of the model to aid in preoperative surgical decision making. For predictive modeling, balanced outcome classes (labels) are important to avoid biased learning by the models. To handle class imbalance, up-sampling was carried out to equalize the frequency of the underrepresented class^33^.

### Data pre-processing and predictive modeling

**Figure 7:**
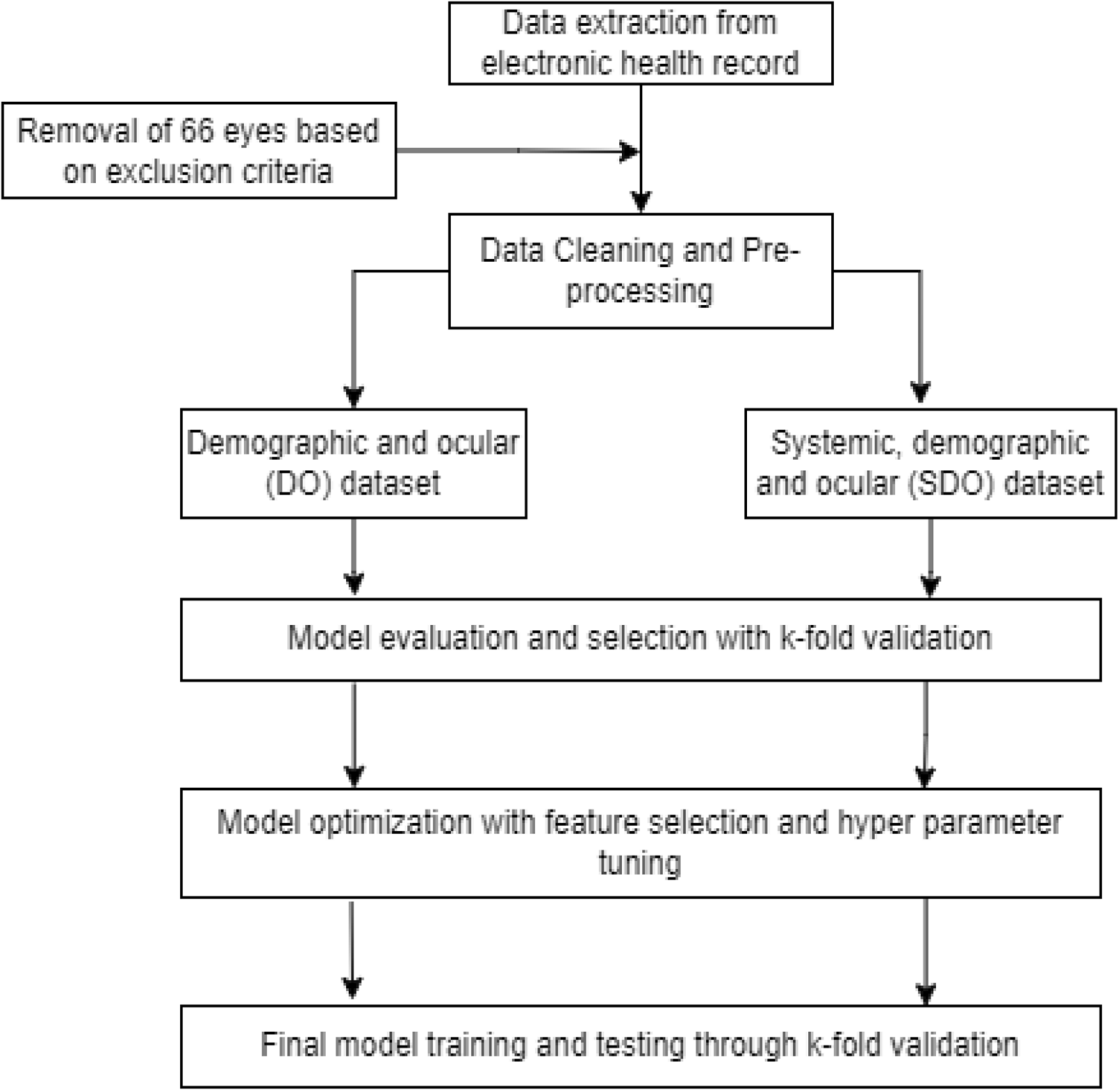
Flow chart of the presented method.

Following data collection, continuous features were normalized with zero mean and unit standard deviation. Additional dummy variables were created when appropriate to obtain grouped categorical features. Visual acuity values were converted into logarithm of the minimum angle of resolution (logMAR). To remove redundancy, pairwise pearson correlation of features was carried out with a threshold of 0.60. For predictive modeling, we exploited random forest, support vector machine, logistic regression and artificial neural network models. We considered two scenarios to determine if the addition of systemic health data improves the predictive outcomes of the models. In the first scenario, systemic health data, demographic and ocular features were considered and in the second scenario, demographic and ocular features were considered. The data were exported into R (version: 3.5.1) for analysis. Summary statistics were generated to describe both success and failure cohorts. Univariate analysis was performed to investigate potential associations between demographic, systemic health and ocular features individually with the primary outcome. Figure 7 depicts the overall workflow for data collection, data processing, model construction and evaluation.

*Random forest* predicts outcomes by relying on the outcomes of an ensemble of individual decision trees, which are trained on subsets of the data. Each decision tree uses multiple thresholds to simplify the complex decision process into a collection of decision boundaries. The learning process initiates at each internal node where feature observations result in two sub-branches depending on the outcome of a decision rule. This learning process recursively splits observations set into two subsets, following the decision rule, eventually resulting in a terminal node that represents the corresponding class of the observation^34^. Features are randomly selected as candidates for the split at each node, where the number for these features is much smaller than the total number for available features. Random forest formed this way, with random input selection is called Forest-RI^35^. Random forest predictive model reduces the risk of over-fitting by averaging over the decisions of multiple decision trees. We utilized the ‘randomForest’ package from R to implement this model using 500 trees (“ntree”), the number of variables randomly sampled at each split (“mtry”) set to the default of the square root of the number of features, and the minimum size of of terminal nodes (“nodesize”) set to one^36^.

*Multivariable logistic regression* is a commonly used model for outcome predictive modeling. It determines the outcome of the observation by multiplying each feature with a constant, adding a bias term and applying a logistic function^37^. The outcome of the model is rounded to 1 if it is above 0.5 and considers 0 if below 0.5. The value of the bias terms is estimated to fit the data using the maximum likelihood method. With that estimate, the probability is calculated for new data point to classify it based on the threshold. We used accuracy to add or remove variables from the model starting from an empty (null) model. We adopted a bidirectional variable selection using the ‘caret’ package from R for this model^38^.

*Artificial neural networks (ANN)* leverage input features that are connected with a neuron which multiplies them with a weight, adds an intercept and applies a subsequent function to generate the output. It imitates neural tissues by sequentially transforming the features and passing them on to the next layer. The final layer of the network combines the output by extracting the information from these neurons. We compared different neural network architectures, varying number of layers and neurons in each layer, and found no considerable difference in terms of performance. For each neural network architecture, the maximum iteration variable was set to 100 epochs. Eventually, we utilized the default neural network architecture from the package ‘neuralnet’ in R with one intrinsic layer and one neuron, since further optimization was not in consideration during model selection phase.

*Support vector machine (SVM)* uses support vectors to identify hyperplanes with maximum separable margin to further predict the class of the observation. A non-linear mapping transforms original observations into high dimensional space observations using a kernel function. Such multi-dimensional mapping helps in finding a linear or non-linear decision boundary (hyperplanes) in the transformed observation space while maximizing the margin of separability between different classes. The structure of the hyperplanes depends on the type of kernel function. A decision function then distinguishes transformed high dimensional observation by defining optimal separable hyperplanes. We used the ‘e1071’ package from R to implement our SVM model, using a radial kernel function with a default gamma of 1/(number of features), C-constant (cost) of 1, and epsilon of 0.1^39^.

### Validation and performance analysis of predictive models

Cross validation is primarily used to estimate the performance of the predictive models and to avoid over fitting. We used a k-fold cross-validation scheme with $k=5$^40,41^. The validation data set is divided into k folds or groups of approximately equal size data sets. Folds of $k-1$ are used to train the predictive model and the trained model is tested on the remaining 1 fold. This procedure is repeated k times allowing for each fold to be tested against the remaining $k-1$ folds. The overall predictive performance is determined by aggregating the performance of all validation fold groups. We evaluated four metrics to analyze predictive performance: accuracy, sensitivity, specificity and AUC. The model with the best performance was selected for further recursive feature selection and hyper-parameter tuning. Improvement in surgical outcome prediction accuracy was used as a measure to select the optimal features from each dataset scenario. The set of features that produced the highest accuracy was selected for model training/testing and the remaining features were eliminated. Following feature selection, the best performing model in terms of accuracy was chosen from the entire grid of generated models. Calibration of the final SDO and DO random forest models were analyzed using a calibration plot^30^. The calibration slope, with an optimal value of 1, was calculated to assess whether predictions were precise or too extreme. Calibration-in-the-large, or y-intercept of the calibration plot, indicating the degree to which predictions are systematically too low or high, was calculated, having an optical value of 0. A decision curve analysis (DCA) was used to evaluate the clinical usefulness of the final SDO and DO random forest models by calculating their net benefit across a range of clinical threshold probabilities^42,43^. An advantage of DCA is that it incorporates preferences (patient and physician), represented as threshold probability of choosing or opting out of a treatment, across a range of probabilities. Ranked feature importance using the mean decrease in accuracy (MDA) and mean decrease in importance (MDI or Gini Importance) methods was performed on this final model. All source code is available for public use on Github at https://github.com/HasanulbannaR/ML_Trab.git.
